# Higher speciation and lower extinction rates influence mammal diversity gradients in Asia

**DOI:** 10.1186/s12862-015-0289-1

**Published:** 2015-02-04

**Authors:** Krishnapriya Tamma, Uma Ramakrishnan

**Affiliations:** National Centre for Biological Sciences, TIFR, Bellary Road, Bangalore – 65, India

**Keywords:** Biomes, Biogeography, Tropical diversity, Diversification rates, Latitudinal gradients, Phylogenetic diversity

## Abstract

**Background:**

Little is known about the patterns and correlates of mammal diversity gradients in Asia. In this study, we examine patterns of species distributions and phylogenetic diversity in Asia and investigate if the observed diversity patterns are associated with differences in diversification rates between the tropical and non-tropical regions. We used species distribution maps and phylogenetic trees to generate species and phylogenetic diversity measures for 1**°** × 1**°** cells across mainland Asia. We constructed lineage-through-time plots and estimated diversification shift-times to examine the temporal patterns of diversifications across orders. Finally, we tested if the observed gradients in Asia could be associated with geographical differences in diversification rates across the tropical and non-tropical biomes. We estimated speciation, extinction and dispersal rates across these two regions for mammals, both globally and for Asian mammals.

**Results:**

Our results demonstrate strong latitudinal and longitudinal gradients of species and phylogenetic diversity with Southeast Asia and the Himalayas showing highest diversity. Importantly, our results demonstrate that differences in diversification (speciation, extinction and dispersal) rates between the tropical and the non-tropical biomes influence the observed diversity gradients globally and in Asia. For the first time, we demonstrate that Asian tropics act as both cradles and museums of mammalian diversity.

**Conclusions:**

Temporal and spatial variation in diversification rates across different lineages of mammals is an important correlate of species diversity gradients observed in Asia.

**Electronic supplementary material:**

The online version of this article (doi:10.1186/s12862-015-0289-1) contains supplementary material, which is available to authorized users.

## Background

The latitudinal gradient in species diversity is globally pervasive [1,2] and several ecological (e.g., climate) and evolutionary (e.g., niche conservatism, diversification rate heterogeneity) hypotheses have been proposed to explain this gradient [3,4]. In many taxa, these gradients are correlated with both past and contemporary climate [5,6] although the mechanisms driving these correlations are not clearly understood [7]. The relationship between diversity and environment is not the same across species pools [6,8] owing to differences in biogeography and evolutionary histories.

But how are these diversity gradients established? Speciation and extinction (diversification) ultimately drive differences in species richness, and are in turn affected by many evolutionary and ecological factors. Diversification rates can vary across time, across lineages (with some lineages diversifying faster than others) and over space (with some regions diversifying faster than others) [9-11]. As a result, speciation and extinction are inherently historical and stochastic, and can demonstrate environmental and density dependence. By extension, latitudinal gradients in species richness could be explained by the tropics having higher speciation rates compared to temperate regions [12]. Increased speciation rates in the tropics could be due to greater climatic stability of the region, longer time of existence, their larger area [13], differences in demographic traits correlated with speciation [14] and increased mutation rates in the tropics [15]. Increasing evidence for a large number of taxa suggest that the tropics could be both cradles (lineages originate in the tropics) and museums (lower extinction rates for lineages) of diversity [16,17] and together, these are thought to be the main mechanisms by which such diversity gradients are established.

However, we also know that the correlates of diversity gradients show scale-dependence, that is, the importance of these variables varies across the scales at which diversity is examined. Historical factors including tectonic processes, regional climate and other environmental factors vary across scales and across space. This suggests that both mechanisms and their relative importance in driving speciation and extinction may be different across the tropics as well.

It has been shown that globally, higher speciation and lower extinction rates play important roles in determining the latitudinal mammal diversity gradients [17]. We examine mammal diversity gradients in Asia and ask if spatial variation in diversification rates play an important role in explaining gradients in species richness between tropical and non-tropical regions here.

Situated between Europe, North America, and Africa, tropical Asia is one of the world’s most biodiverse regions and has exchanged mammals with these adjacent areas multiple times in the past [18,19,20]. Recent fossil discoveries reveal the presence of some of the oldest placental mammal fossils in Asia [21], pointing to an important role for this region during early placental mammal evolution. However, little is known about how mammal lineages have diversified subsequently here. The great climatic and habitat diversity found here provide a unique opportunity to study diversification patterns across a broad spectrum of clades and biomes. Biogeographically, Asia displays a pronounced north–south divide (due to the Tibet-Himalaya barrier), with the Palearctic realm occupying all of the northern part of the continent. Only a few continental scale analyses of diversity patterns exist for this region (see [22]) and we know little about mammalian diversity gradients and their correlates in mainland Asia and how they compare to those determined globally.

**Figure 1:**
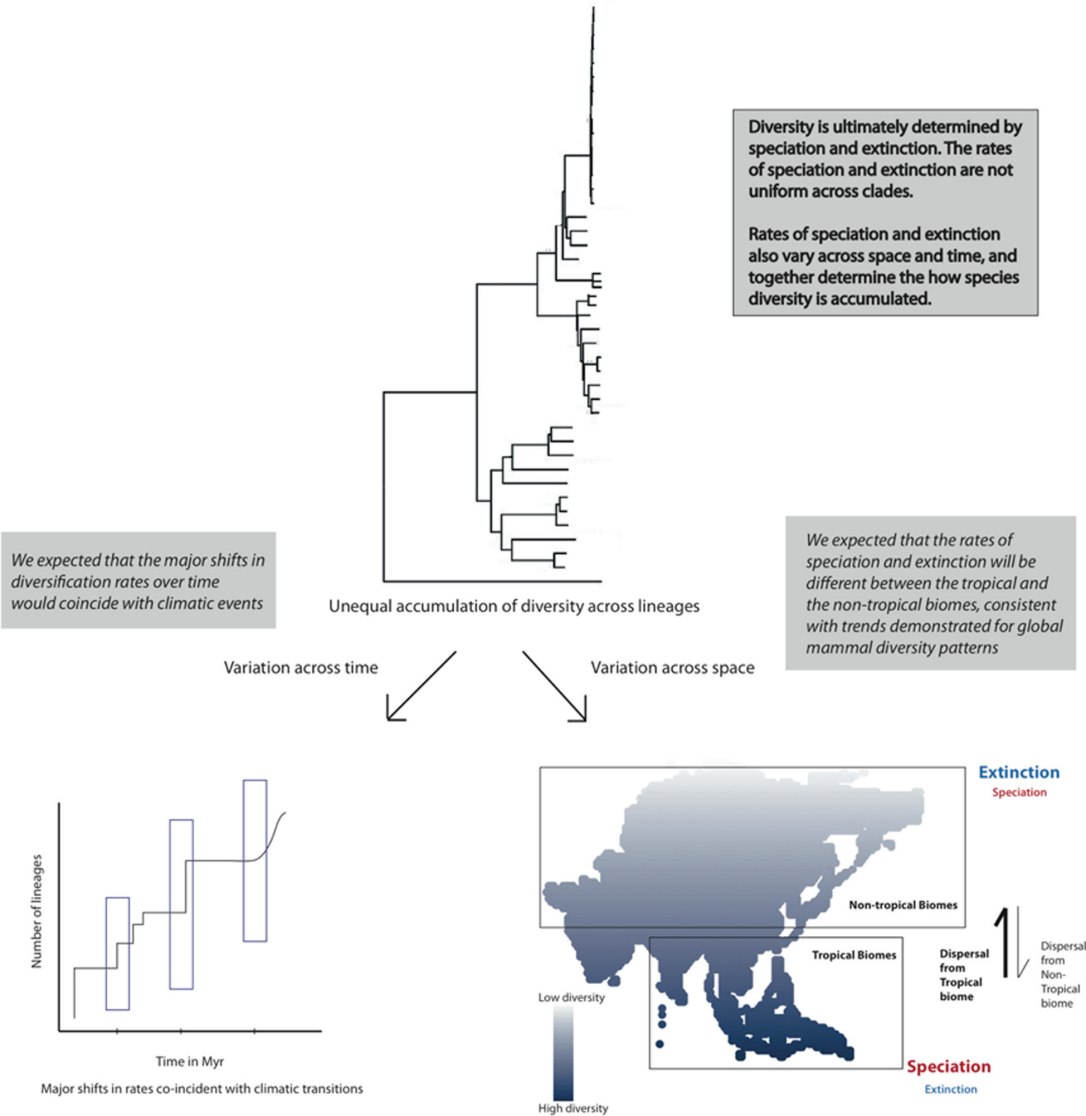
**An illustrative figure outlining the expectations.** The size of the font and the arrows indicates the strength of the effects (darker, bolder, thicker indicates stronger effects, while lighter, and thinner indicates weaker effects).

In this paper, we first demonstrate the presence of latitudinal and longitudinal gradients in species diversity in mainland Asia. We explore the relationship between species richness and phylogenetic diversity for different orders to ask if there are differences in the evolutionary histories of lineages across Asia. We then examine if differences in diversification rates could correlate with observed patterns in diversity distribution. For that, we examine if diversification rates vary across time and space for mammals in Asia using lineage through time plots and likelihood functions. Finally, we test if the observed diversity gradients could be associated with spatial variation in diversification rates across mainland Asia, using biome-level analysis. Similar to patterns recovered at a global scale [23], we expect diversification rates to vary across the tropical and temperate regions in Asia resulting in the observed diversity patterns (Figure 1). In particular, we use novel approaches applied at the biome level to estimate how speciation, extinction and dispersal together contribute to differences in diversity across mainland Asia.

## Methods

### Geographical extent

We analyzed mammal diversity patterns for Asia, and used the political definition of Asia for the same.

### Mammalian orders

To investigate if trends are congruent across different lineages, all our Asia- level analyses were conducted for all mammals considered together, and for each of the following orders – Artiodactyla (even-toed ungulates), Carnivora, Rodentia (rodents), Chiroptera (bats), Lagomorpha (rabbits and pikas) and Soricomorpha (shrews).

### 1. Latitudinal gradients in diversity

#### Spatial dataset

We used geographical range-maps for 1863 mammalian species (457 genera, Additional file 1) from IUCN [24] to derive the number and identity of species in 110 km × 110 km (1**°** × 1°) grid cells across Asia. To ensure that all cells represented similar areas (irrespective of their latitudinal position), we used Lambert’s equal area projections. All spatial analyses were carried out in QGIS v. 1.8.0 [25]. Many of our analyses were conducted at the scales of biomes (derived from [26]). Biomes are natural blocks of environment and habitat with distinct evolutionary trajectories. Many of the biomes have distinct evolutionary histories (such as expansion or contraction in response to climate) that may have impacted the species that are restricted to them. Further, the evolution of many biomes could have influenced the evolution of animals associated with the biomes (for example, even-toed ungulates and savannas [27]).

#### Species richness

A species was recorded as being present in a cell if any part of its distribution overlapped with that cell. This criterion may err on the side of overestimating ranges but since the IUCN ranges are crude estimates, we believe that our criterion will not bias our results. The total number of overlapping ranges in a cell was taken as the measure of species richness for that cell. Species by site (community) matrices were created with rows denoting cells/biomes and the columns denoting species.

Species richness is an insufficient metric of diversity, as it does not include species identities or their evolutionary histories [28]. We used Phylogenetic diversity (PD) to incorporate evolutionary relationships among species into the assessment of diversity. Evolutionary relationships were derived from published mammal supertrees.

#### Phylogenetic diversity

We based our analyses on a phylogenetic tree that was built by resolving polytomies of the completed and re-dated phylogeny of Bininda-Emonds [29] as used in [17]. This phylogenetic tree accounts for most of the global mammal diversity (representing 5020/5416 species). We henceforth refer to this tree as the ‘global-tree’.

Faith’s PD (sum of the branch lengths of the phylogenetic tree describing the evolutionary relationships of the members of a community) was calculated for each cell using the global-tree. PD was also calculated for each major mammalian order. To determine if there are differences in the evolutionary histories of the different orders across space, the relationship between PD and SR was examined. We built regression models between SR and PD for each of our orders and tested for both linear and quadratic relationships. Spatial autocorrelation was included as a smooth term in a generalized additive model [30]. From the model, we obtained the residuals and identified the top and bottom 10% of all residuals [31]. These represent greatest deviations from the global relationship between PD and SR. Thus, they represent cells with the unusually high or low PD; these cells were identified and plotted.

### 2. Temporal patterns in diversification rates of lineages

#### Phylogenetic data

To determine temporal patterns in diversification, we used a phylogenetic tree representing 457 genera of mammals, chosen as they are predominantly distributed in Asia. This tree, based on the mammal supertree described above, is thus representative of lineages that are predominantly distributed in Asia and that are impacted by processes occurring in this region.

#### Lineages-through-time plots

Lineages-through-time (LTT) plots are graphical representations of the accumulation of species over time, and are derived from dated phylogenies [29]. We constructed LTT plots from our phylogenetic tree for all mammals across Asia and for each mammalian order. LTT plots are affected by the ‘pull of the present’ [32], so we did not estimate diversification rates from these plots, using instead likelihood approaches like the one described below (see Additional file 1).

#### Diversification shift times

To determine the time periods associated with significant changes in diversification rates, we used the maximum likelihood method as introduced and described by Stadler [32]. This method uses a maximum likelihood function to estimate the number of significant changes (shifts) in speciation and extinction rates, and the time periods between these shifts from a given phylogenetic tree. We employed this method on the tree for all Asian mammals, and for each order. Incorporating sampling frequencies into the models controlled for incomplete sampling of our phylogenies. The sampling frequencies were calculated (for each genus) as the ratio of the number of species represented in the tree to the total number of species in the lineage. We built models to obtain the estimates for zero, one, two, three, four and five diversification rate changes (shifts) for all Asian mammals and each of the orders. Likelihood ratio tests (of models with shift compared to shift + 1) were used to determine the best-supported shift times. The analysis was also conducted for the tree from [33], since the two trees differ in the dates.

### 3. Geographical variation in diversification rates

#### Phylogenetic data

To determine spatial patterns in diversification rates at the global level, we used the mammal supertree (global-tree). For spatial patterns in diversification rates in Asia, we used a phylogenetic tree derived from the global-tree that retained lineages distributed in Asia. This tree was generated by pruning the global-tree to contain all descendants of genera that are reported from Asia (henceforth referred to as Asia-tree). Thus, we used data for 457 genera that were predominantly distributed in Asia (Additional file 1).

#### Global analyses

Disentangling the roles of speciation and extinction in driving diversity gradients is now possible with the availability of large well-resolved phylogenies and analytical tools. The Geographic State Speciation and Extinction (GeoSSE) models the reciprocal interaction between the geographic range evolution and the increase in species diversity [34]. This method uses likelihood approaches to estimate region-dependent rates of speciation, extinction and dispersal using phylogeny and geographical state information [12,35]. The geographical information consists of three states – A, B (species endemic to a single region, A or B) and AB (species widespread between both regions). Speciation can occur in region A (*sA*), B (*sB*). sAB refers to the between region mode of speciation wherein a species can undergo speciation to give rise to daughter species, one in each region. Geographical range evolution can occur by range expansion or range contraction. Geographical range expansion occurs from A or B to AB with the rates of dA and dB. Geographical range contraction occurs via extinction, with species going extinct in A or in B with rates of ×A and ×B (See [34] for more details).

Instead of latitudinal bands, we contrasted diversification (speciation, extinction and dispersal) rates across the tropical biomes and the non-tropical biomes. The tropical and subtropical moist broadleaf forests (B1); tropical and subtropical dry broadleaf forests (B2); tropical, subtropical coniferous forests (B3) and tropical and subtropical grasslands, savannas and shrublands (B7) were designated as ‘tropical biomes’ and all the other biomes as ‘non-tropical biomes’. We used this biome classification instead of latitude to explore variation in diversification rates since these biomes are largely found in the tropical band and show highest diversity. The mammalian distribution data from IUCN was intersected with the map of the biome distribution, and geographic state information for each species was derived. If a species range was restricted within the tropical biomes, its geographical state was coded as 1, if its range was restricted to the non-tropical biomes, its geographical state was coded as 2, and if it was found in both biomes, its geographical state was coded as 0. Thus, the geographical states of 1, 2, and 0 represent species found in tropical regions, species found in non-tropical regions and species found in both regions.

The GeoSSE algorithm can account for missing species by incorporating the sampling frequency of the phylogeny in the likelihood estimations. We estimated the sampling frequencies to be as follows: 0.66 for species spanning both biome types, 0.747 for species restricted to the tropical biomes and 0.744 for species restricted to the non-tropical biomes (Additional file 1).

We predicted that diversification rates would be different between the ‘tropical’ and the ‘non-tropical’ regions; we tested 16 possible evolutionary scenarios (summarized in detail in Additional file 1) for all mammals globally. These scenarios included constraints on speciation, extinction and dispersal rates across the two regions. For instance compared to the full model wherein all the parameters could vary, scenario 11 constrains sA = sB, ×A = ×B and dA = dB. Thus specific scenarios can be tested. Both likelihood and MCMC approaches were used to estimate the parameters.

#### Asia analyses

We also carried out the GeoSSE analysis for the Asia-tree. Within Asia, we classified the tropical and subtropical moist forest biomes (B1), the tropical and subtropical dry forest biomes (B2) and the tropical and sub tropical coniferous forest biomes (B3) as the ‘tropical biomes’ and all the other biomes as ‘non tropical’ biomes. In Asia the tropical and subtropical grasslands, savannas and shrublands (B7) are not very extensively distributed, and hence were not included in the ‘tropical biomes’. We estimated speciation, extinction and dispersal rates across these two regions for all mammals, and for each of the following orders – Rodentia, Soricomorpha, Chiroptera, Artiodactyla and Carnivora. We cross-verified the trends for Carnivora with a complete phylogeny from Nyakatura *et al.* (Additional file 1). Sampling frequencies were correspondingly calculated to incorporate missing species in our phylogenies. Both likelihood and MCMC based methods were employed to estimate the parameters. Since we found temporal variation in diversification rates, time-dependent scenarios were also tested, and a linear rate of change across all parameters through time was allowed.

We also tested both global- and Asia-level GeoSSE models with re-dated mammalian phylogeny provided by [33] to cross validate our results.

All analyses were carried out in the R statistical environment, v 3.0 [36]. Functions from the package ‘picante’ [37] were used to calculate PD and to prune trees. LTT-plots were constructed using the package ‘ape’ [38], and abrupt changes in diversification (and their timing) were estimated using functions in package ‘TreePar [32]. GeoSSE was performed using function from the package ‘diversitree’ [39].

## Results

### Tropical Asia is both species-rich and phylogenetically diverse

#### Species richness

**Figure 2:**
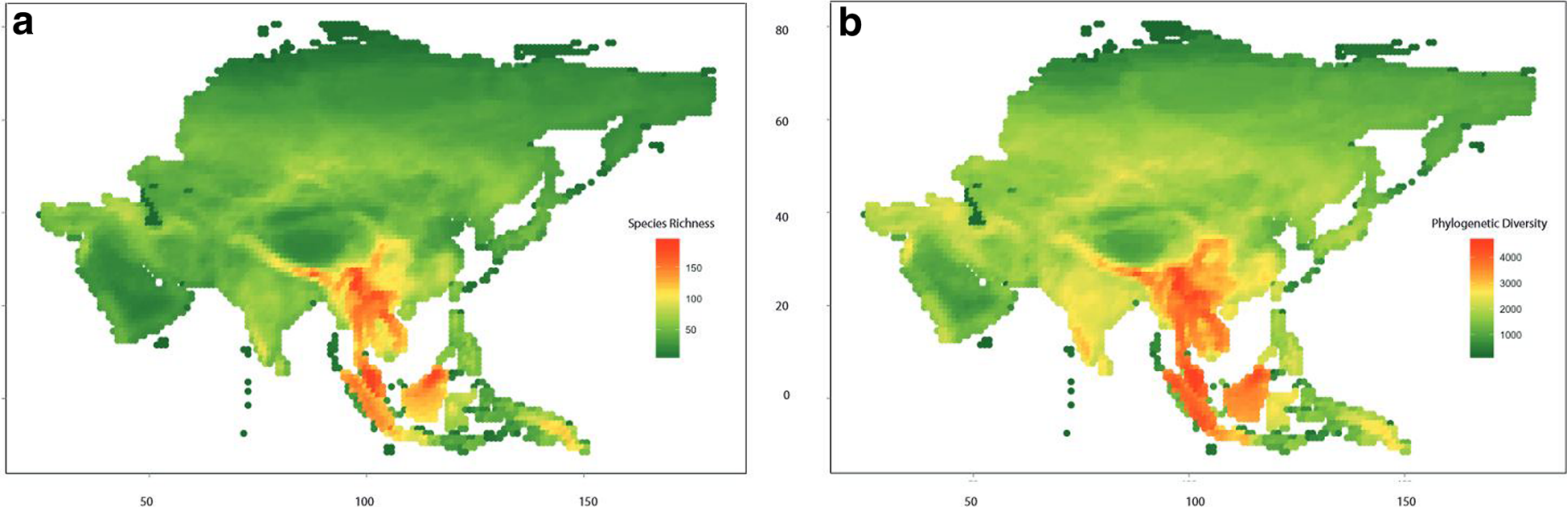
**Spatial distribution of mammal species and phylogenetic diversity in Asia. (a)** Species richness (SR) and **(b)** Phylogenetic diversity (PD) in 1° × 1° cells across Asia. In both figures red corresponds to high species diversity and green corresponds to low species diversity.

Mammal species richness was highest in tropical South Asia, particularly in Southeast Asia and the Himalayas, with richness of ca. 200 species per cell (Figure 2a). Mammalian species richness (SR) generally followed a latitudinal gradient with the lower latitudes being more diverse than the higher latitudes. Additionally, within the low latitudinal band, we also observed a longitudinal gradient in species richness, with higher species richness in eastern Asia. The tropical moist forest biome (B1) had the highest species richness, and the tundra (B11) the lowest richness amongst the relatively common biomes in Asia (Table 1).

**Table 1:** **The different biomes in Asia and the number of mammal species in each**

**ID**	**Biome**	**Species richness**
B1	Tropical and subtropical Moist broadleaf forests	1418
B2	Tropical and subtropical Dry broadleaf forests	478
B3	Tropical and subtropical coniferous forests	481
B4	Temperate broadleaf and mixed forests	731
B5	Temperate coniferous forests	726
B6	Boreal forests/Taiga	159
B7	Tropical and subtropical grasslands, Savannas and shrublands	307
B8	Temperate grasslands, Savannas and Shrublands	395
B9	Flooded grasslands and Savannas	236
B10	Montane grasslands and shrublands	976
B11	Tundra	106
B12	Mediterranean forests, woodlands and scrub	147
B13	Deserts and xeric shrublands	608
B14	Mangroves	307

#### Phylogenetic diversity

**Figure 3:**
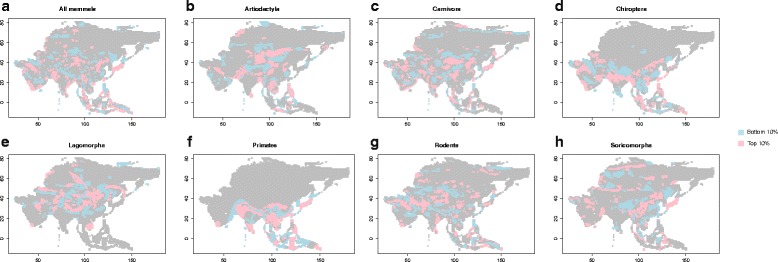
**Spatial distribution of the residuals of the regression (quadratic) between PD and SR, for a) all Mammals, b) Artiodactyla c) Carnivora d) Chiroptera e) Lagomorpha f) Primates g) Rodentia h) Soricomorpha.** The top (pink) and bottom (blue) 10% of the residuals are indicated in this figure.

Phylogenetic diversity (PD) was highest in Southeast Asia and the Himalayas (Figure 2b), and spatial patterns are similar to those of SR. Phylogenetic diversity scales with species richness (r^2^ = 0.985, p < 0.05) (Additional files 1). We examined the regression models between PD and SR for all mammals and each order, and identified cells with higher or lower than expected PD (All mammals - Figure 3a). These cells represent deviations from the expected relationship between PD and SR and thus indicate the impact of evolutionary history on diversity patterns.

For most orders, there are distinct differences in the spatial distribution of the residuals from the regression of PD with SR. For Artiodactyla (Figure 3b) and Lagomorpha (Figure 3e), the highly deviating residuals were largely distributed in the higher latitudes while for Chiroptera (Figure 3d), the highly deviating residuals were largely distributed in lower latitudes. For Rodentia (Figure 3g) and Carnivora (Figure 3c), there seemed to be no distinct pattern across the lower and the higher latitudes.

We also examined the relationship between PD and SR across different biomes, but the patterns were not very clear for most genera (Additional file 1) except for Rodentia and Chiroptera. Some differences do exist and they confirm the pattern that we observe based on the distribution of residuals.

### Diversification patterns across time

#### LTT plots

**Figure 4:**
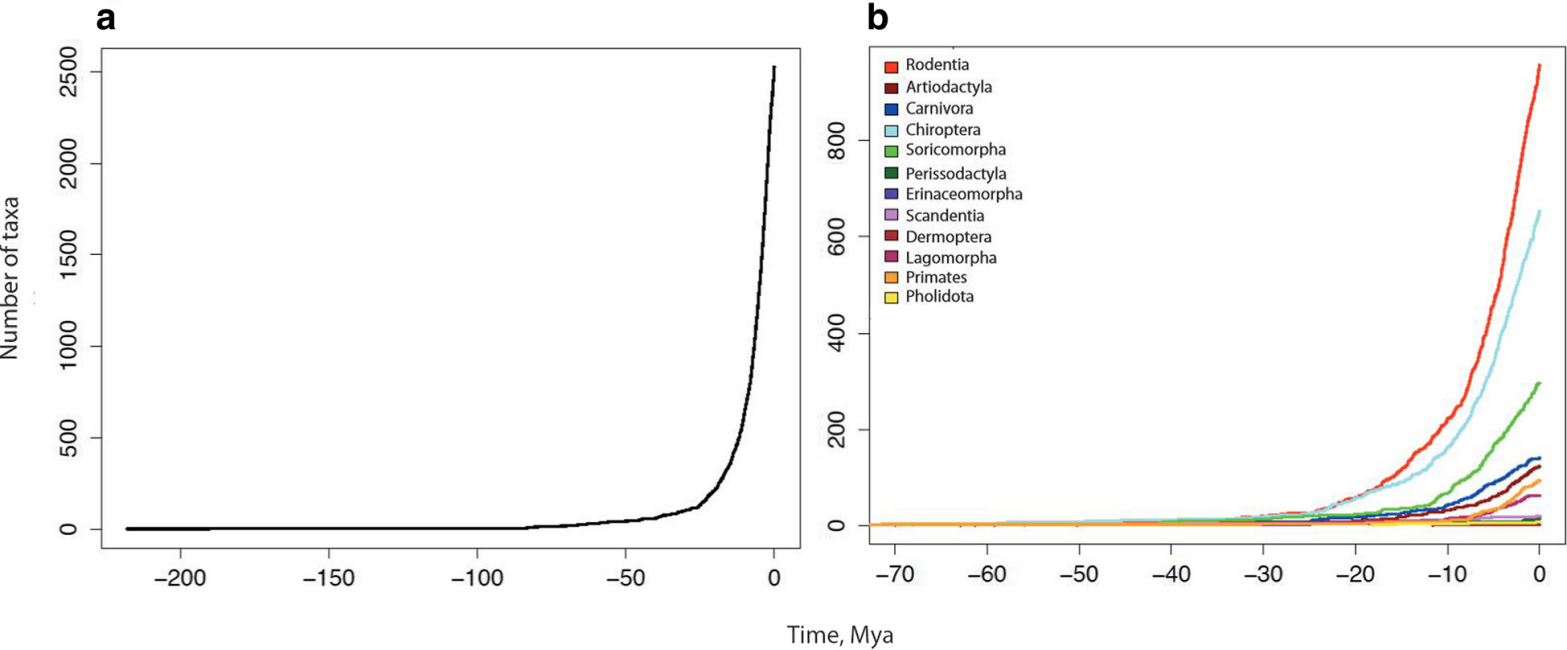
**Lineage-through-time (LTT) plots for (a) all mammals across Asia and (b) all mammal orders in Asia.**

The LTT plots show differences in the rates of accumulation of diversity across time. Figure 4a represents accumulation of diversity for all Asian mammals, Figure 4b represents the major orders found in Asia. Orders with higher species richness (such as Rodentia, Chiroptera and Soricomorpha) show prominent signatures of changing diversification rates, with a steep rise in the number of taxa within the last ca. 20 million years, while plots for less speciose groups such as Primates and Perissodactyls show a shallower slope, implying less drastic changes (See Additional file 1 for trends of each order).

#### Temporal shifts in diversification rates

We estimated temporal variation in speciation, extinction rates and diversification rate changes for all Asian mammals combined together. We detected three significant diversification rate shifts throughout the history of the lineage (Table 2). An increase in diversification rates at 80 Mya lasted until 10 Mya, followed by a slight decline lasting till 5 Mya. At ca. 5 Mya, the diversification rates in Asian mammals increased.

**Table 2:** **Estimates of best-supported shift-times for all Asian mammals and each order**

**Taxon group**	**Species richness**	**Turnover**	**Diversification rate**	**Shift time (Mya)**	**Number of shifts**
**All Asian Mammals**	2549	1.25	−2.5×10^−2^		3
		8.5×10^−1^	3.9×10^−2^	80	
		8.08×10^−1^	3.50×10^−2^	10	
		2.7×10^−7^	1.25×10^−1^	5	
**Rodent**	959	1.46×10^−1^	5.27×10^−2^		1
		6.09×10^−7^	1.41×10^−1^	25	
**Artiodactyla**	126	3.51×10^−6^	2.01×10^−2^		1
		1.63×10^−1^	1.17×10^−1^	30	
**Chiroptera**	657	2.96×10^−1^	4.32×10^−2^		1
		6.85×10^−7^	1.29×10^−1^	25	
**Soricomorpha**	298	8.28×10^−1^	4.23×10^−2^		1
		6.41×10^−7^	1.14×10^−1^	5	
**Lagomorpha**	64	9.77×10^−1^	5.85×10^−3^	5	1
		7.95×10^−8^	1.25×10^−1^		
**Carnivora**	159	1.31×10^−6^	3.71×10^−2^		1
		3.92×10^−6^	1.04×10^−1^	25	
**Primate**	95	9.97×10^−1^	1.0×10^−4^		1
		9.73×10^−2^	1.7×10^−1^	5	
**Erinaceomorpha**	19	4.37×10^−6^	4.57×10^−2^	-	0
**Pholidota**	9	3.48×10^−7^	6.10×10^−2^	-	0
**Scandentia**	21	4.37×10^−6^	4.57×10^−2^	-	0
**Perissodactyla**	15	1.02	−0.002	-	0

Turnover = Extinction rate/Speciation rate; Diversification = Speciation – Extinction. Shift time corresponds to the time when the significant shift was detected, and the number of shifts is denoted.

Additionally, we also estimated diversification rate for each mammalian order. For Rodentia, Carnivora and Chiroptera, we detected one significant change in diversification rate at 25 Mya, Artiodactyla at 30 Mya, Soricomorpha, Lagomorpha and Primates at 5 Mya (Table 2). Four orders, Perissodacyla, Pholidota, Erinaceomorpha and Scandentia showed no significant change in their diversification rates over lineage history.

Analyses of diversification rate change were slightly different across the two trees, but largely conform to showing shifts at 30 Mya, 25 Mya and 5 Mya, with additional shifts at 40 Mya for Rodentia and Lagomorpha (Additional file 1).

### Diversification rates differ across the tropical and non-tropical biomes

#### Global analyses

**Figure 5:**
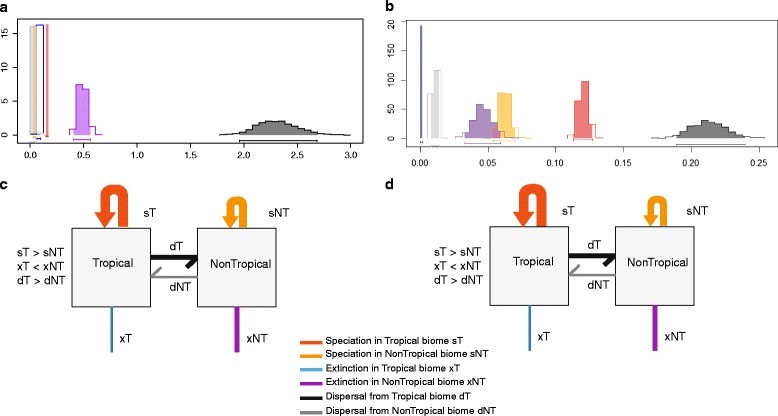
**Distribution of the MCMC derived estimates for speciation, extinction and dispersal rates across the a) Global and b) Asia biomes.** Schematic showing the same for **c)** Global and **d)** Asia with the boxes representing the tropical and non-tropical regions. The colors are the same across the two panels.

We estimated speciation rates, extinction rates and dispersal rates from the two regions – tropical and non-tropical biomes (GeoSSE models). We find that diversification rates vary between the ‘tropical’ and the ‘non-tropical’ biomes. We tested 16 different scenarios (models) using both likelihood and MCMC approaches and our results were consistent across the two approaches. The results for the 16 scenarios are summarized in the Additional file 1. AIC was used for model selection. The best model estimated speciation rates to be higher in the tropical biomes than the non-tropical biomes, extinction rates to be lower in the tropical biomes and dispersal rates to be higher from the tropical to the non-tropical biomes (Figure 5, Table 3).

**Table 3:** **Speciation, extinction and dispersal rate estimates (model with best support) for the tropical and non-tropical biomes for all global mammals, all Asian mammals and each of the orders**

**Dataset**	**Estimation method**	**Temporal**	**sT**	**sNT**	**sT-NT**	**xT**	**xNT**	**dT**	**dNT**
All Global Mammals	Likelihood	Time constant	0.15	0.0413	0	0.076	0.510	2.73	1.52×10^−3^
All Global Mammals	MCMC	Time constant	0.159	0.04	0	0.089	0.5	2.4	0.039
All Asian mammals	Likelihood	Time Constant	0.13	0.0622	0	1.16×10^−5^	0.039	0.21	0.012
All Asian mammals	Likelihood	Time dependent	0.136	0.029	0	2.9×10^−8^	0.094	2.9×10^−7^	6.2×10^−3^
All Asian mammals	Likelihood	Slope of parameters	sA.m = −6.22×10^−4^	sB.m = 2.19×10^−2^	0	×A.m = 2.48×10^−3^	×B.m = 2.15×10^−2^	dA.m = −1.35×10^−3^	dB.m = −1.43×10^−5^
All Asian mammals	MCMC	Time constant	0.129	0.066	0	5.5×10^−4^	0.047	0.219	0.011
Rodents	Likelihood	Time constant	0.15	0.0919	0	4.71×10^−9^	0.04	0.17	9.3×10^−3^
Soricomorphs	Likelihood	Time constant	0.13	0.062	4.1×10^−3^	7.4×10^−9^	5.2×10^−9^	0.12	0.0189
Chiroptera	Likelihood	Time constant	0.16	0.046	0	0.076	0.449	0.98	2.23×10^−7^
Carnivora	Likelihood	Time constant	0.07	0.07	0	0.23	0.21	3.91	1.008
	Likelihood	Time constant	0.07	0.07	0	0.61	0.14	2.64	2.64
	Likelihood	Time constant	0.07	0.07	0	0.22	0.22	4.25	0.96
Artiodactyla	Likelihood	Time constant	0.088	0.12	0	0.067	2.12×10^−8^	0.017	0.26
	Likelihood	Time constant	0.117	0.117	0	0.102	9.137×10^−8^	0.0154	0.291
	Likelihood	Time constant	0.0649	0.127	0	2.25×10^−9^	2.25×10^−9^	0.0292	0.145

The estimation method is either of likelihood or MCMC, and temporal refers to time constant or time-dependent estimation. The estimates are for:

sT – Speciation rate in Tropical biomes.

sNT – Speciation rate in Non tropical biomes.

sT-NT – Between region speciation rate.

xT – Extinction rate in Tropical Biomes.

xNT – Extinction rate in Non Tropical biomes.

dT – Dispersal from tropical biomes.

dNT – Dispersal from Non tropical biomes.

#### Asia analyses

We also estimated speciation, extinction and dispersal rates for the Asia-tree across the tropical and non-tropical biomes in Asia. We found a similar pattern with higher speciation rates, lower extinction rates in the tropical biomes (Figure 5, Table 3) (See Additional file 1 for all scenarios). When we ran the analyses for five mammalian orders individually, the orders differed in the rates of diversification across tropical and non-tropical biomes. Rodentia and Chiroptera showed higher speciation and lower extinction in tropical biomes (Table 3). Soricomorpha revealed higher speciation in tropical biomes, but equal extinction rates across the two regions. For Carnivora, we recovered equal rates of speciation and extinction in tropical and non-tropical biomes. Analyses for Artiodacyla revealed equal rates of speciation but higher rates of extinction in tropical biomes. However, for Carnivora and Artiodactyla, three models were equally supported (ΔAIC < 2, Table 3).

Since our lineages also show changes in diversification rates across time, we incorporated this into our Asia-level analyses of GeoSSE models as well. The time-dependent models were run with the same data, after allowing for linear rate of change across all parameters through time (Table 3). The estimated parameters from this time-dependent analysis also suggest that tropical biomes show higher speciation and lower extinction than the non-tropical biomes. We did not run the time-dependent models for the global dataset – since our results corroborated with that of [17] who have performed an extensive analysis of mammal diversification rates at the global level.

Overall, all results were consistent across the two trees tested (global-tree, and from [33]), both for the global and the Asian dataset (Additional file 1).

## Discussion

Our study sought to explore evolutionary causes of the mammalian species diversity patterns in Asia. We find that the observed species and phylogenetic diversity gradients could arise due to differences in the evolutionary histories of lineages across space and time. The processes of speciation, extinction and dispersal largely drive differences in evolutionary histories (and hence composition of regional pools). Importantly, we demonstrate that differences in diversification rates across tropical and non-tropical biomes could be important correlates of the diversity differences between them.

### Mammal diversity patterns in mainland Asia

Mammal diversity patterns in Asia reveal a strong latitudinal gradient, as expected from global patterns and those for other taxa in Asia [22]. Tropical regions harbor more species and phylogenetic diversity than the temperate regions. Globally, tropical South America and tropical Africa (especially the rift valley) show similar patterns [4]. However, in mainland Asia, the diversity is not uniformly high across the tropical latitudinal band, with a longitudinal gradient in both species and phylogenetic diversity as well. For instance, the Indian subcontinent, despite being a part of the tropical latitudinal belt and proximal to Southeast Asia, shows a very unambiguous decrease in species and phylogenetic diversity. The Indian subcontinent and the regions to the west of it show increasingly arid regimes, correlating with the observed longitudinal gradient. Both regions remain poorly surveyed and sampling biases may not contribute significantly to this pattern.

Phylogenetic diversity (PD) scales with species richness (SR) and the non-linearity of the relationship has been demonstrated before [28]. Deviations from the expected relationship between PD and SR represent differences in the evolutionary histories of the communities, associated with differences in the processes by which diversity is accumulated (*in situ* speciation or immigration). In our study we detect signatures for the influence of evolutionary history on mammal diversity patterns in Asia (Figure 3). A striking pattern is observed in the Himalayas – which is a dominant mountain chain that separates the Palearctic realm from the Oriental realm. Across orders, the Himalayan regions show deviations from the expected relationship between SR and PD. This could reflect the important role for the Himalayas in influencing diversification processes in Asia, or could also reflect the fact that these are regions where taxa from two biogeographic realms (Palaearctic and Oriental) meet – resulting in the distinct patterns observed in phylogenetic diversity [31]. The Western Ghats – a prominent mountain chain along the west coast of India - also shows deviations from expected PD-SR relationship. The Western Ghats have many endemic species, and several of these have sister species in peninsular India (e.g., *Mus famulus*, *Macaca silenus*).

Though islands generally could be predicted to show lower PD than expected (due to isolation followed by *in situ* speciation), we did not find any consistent pattern in island Southeast Asia. One of the reasons could be the cycles of connection and isolation with the mainland due to sea level changes during the Pleistocene glaciations. Inadequate sampling of the islands, coupled with cryptic speciation, could contribute to the lack of expected pattern; these are issues that are relevant in the mainland as well.

The spatial distribution of residuals largely concurred with known patterns for Lagomorpha. Lagomorphs reach high diversity in the mid-latitudes, especially in the Tibetan plateau (Additional file 1). Further, many species of lagomorphs became extinct during the Pleistocene [40]. On the other hand, Chiroptera shows most deviation from the relationship between PD and SR in tropical Asia where its richness also peaks (Southeast Asia and the Himalayas) (Additional file 1). This suggests that this region, especially along the Himalayas and in Southeast Asia, shows distinct patterns of diversity assembly driven either by speciation (in regions where PD is lower than expected) or immigration (for instance, in regions where PD is higher than expected).

Despite being largely restricted to tropical latitudes of Asia, Primates too show distinct patterns in the spatial distribution of the residuals with regions along (and surrounding) the Himalayas showing higher than expected PD. The Himalayas themselves show lower than expected PD which suggests that the Himalayas may be accumulating species by processes that are very different from regions surrounding them [41].

Thus PD for mammals in Asia reveals a complex history of diversification across regions for the different orders. It also suggests that the process of assembly of mammal diversity is very different across regions, either driven by differences in diversification rates themselves or in dispersal and successful establishment.

There are 75 Asian species missing from the mammal supertree (see Additional file 1). Most of these species are distributed in Southwest China and Southeast Asia (Additional file 1), regions that are already characterized by high PD. Moreover, there is potentially a lot of unrecognized and unknown diversity in Asia, particularly in tropical Asia. Since there are more chances of discovering unknown diversity in the tropics [42], we believe that further additions to diversity will only strengthen the patterns (of high tropical diversity and high diversification rates) recovered by the study (See Additional file 1).

### Mammal diversification rates have changed over time

An analysis of global mammal phylogeny revealed four significant diversification rate changes – at 33, 30, 8.55 and 3.35 Mya [32]. Asian mammals show significant changes in diversification rates at ca. 80 Mya, 10 Mya and 5 Mya. Asia or its mammals did not exist in the current conformation 80 Mya and hence we restrict our inferences to the time period post Eocene epoch. There is broad concordance in the diversification times for Asian mammals and the global patterns for this period, though we did not recover a shift at 33–30 Mya. For these data, the model with 4 rate changes shows a diversification rate change at 25 Mya. The difference in the likelihood values for these two models (change = 3 and change = 4) is not significant at p = 0.05. However, when we estimated shift times for individual orders, we recovered shifts at 25 Mya for Rodentia, Carnivora and Chiroptera (see below).

The timing of the origin of mammalian diversity and its subsequent evolution has been debated for long ([43,29]). Significant changes in mammal diversification rates in Asia only occurred more recently – 10 Mya and 5 Mya though the diversification rate change at 10 Mya is not very large. Apart from corresponding to the Miocene-Pliocene transition that is largely associated with cooling and aridification, this time period (ca. 10 Mya) also corresponds to the uplift of the Tibetan plateau (see below).

Artiodactyls show a significant rate change about 30 Mya probably associated with the Ecocene-Oligocene transition and the evolution of grassland biomes. Rodentia, Carnivora and Chiroptera show an increase in diversification around 25 Mya. Davies et al. [44] found that diversification rate changes for flowering plants was highest around 25–40 Mya, which could result in an increase in diversification of bats, especially frugivorous ones [45]. Many hyaenodontids (Carnivora) were present in Asia and Africa during the Oligocene. The evolution of many primitive canids and felids could be detected as an abrupt diversification rate change by the analysis. In Asia, stratigraphic evidence suggests that this period (Oligocene- Eocene transition, between 30 and 20 Mya) was associated with increasing aridity and cooling [46] and turnover in mammal communities (Mongolian remodelling) [47]. Further shifts to aridity are also observed from paleoclimatic reconstruction and stratigraphic evidence towards the Miocene-Pliocene transition in Asia [48] (Soricomorpha, Lagomorpha and Primates). The concordance in diversification rate changes observed for the individual orders suggests that common correlates, perhaps climate, have impacted their history (“Court Jester” model [49]).

### How has the diversity gradient been established?

Globally, differences in speciation and extinction rates across latitudes have been demonstrated to be important correlates of the observed latitudinal gradient. We find this to be true across the ‘tropical’ and ‘non-tropical’ biomes as well. Overall, we find that the tropics show higher speciation and lower extinction rates than the non-tropics. Further, we also found a similar pattern for Asian mammals with the tropical biomes showing higher speciation and non-tropical biomes showing lower speciation rates. Pruning the mammal supertree generated the Asia-tree. However, the process of retaining certain clades in the tree and removing all others may not result in a random sampling of the phylogenetic tree. This changes the shape of the tree, which may bias our estimates (see Additional file 1 for additional discussion). However, we tested the pattern for tropical biomes at a global scale and demonstrated that they show higher diversification rates. Based on our global results, we are confident in concluding that tropical biomes of Asia may also be associated with higher diversification rates. While we believe that the trends suggested by our Asian-level GeoSSE analysis correctly reflect underlying processes, development of novel analytical frameworks might provide better ability to test these patterns at regional scales. Thus, while the trends suggested by our Asia-level GeoSSE analysis may be reflective of the true patterns, the estimates themselves may not be accurate.

Diversification rates vary amongst lineages either because they are influenced by inherent biological properties of the taxa, or because of interactions between lineages and their environment [50]. Our results add to a growing body of evidence that shows that speciation and extinction rates vary between taxa (due to intrinsic biological factors, such as body size) [14,51], and between geographical regions (tropics and temperate) [52,53]. Additionally, diversification rates may not necessarily change only with latitude, as longitudinal patterns of diversity (especially in Asia) may confound this patterns. For instance, the low latitudinal bands in Asia have regions of very high (Southeast Asia) and very low (west Asia) species richness. Examining patterns across biomes leads to a better understanding of patterns of diversifications, as each biome could be considered an evolutionary unit.

A previous investigation of mammals [54] from across the mammal phylogeny did not find any latitudinal diversification gradient. That study examined diversification rates at the level of genera, which maybe an inadequate scale to investigate such diversity gradients. However, using similar approaches as ours, Rolland and others [17] demonstrated latitudinal gradients in diversification rates globally. Our novel approach of using biomes to assess diversification rates also confirms the gradient of diversification rates from the tropics to temperate regions.

Additionally, when the rates were estimated across the tropical and the non-tropical biomes for different orders, we found order-specific patterns. Rodents, Soricomorpha and Chiroptera showed higher diversification rates in the tropical than the non-tropical biomes. These patterns in diversification rates may be linked to life-history (e.g. body size), demographic or other ecological factors [55]). Historical processes (historical biogeography) may also have an important role in determining species richness patterns [56], and will thus contribute to the lineage-specific patterns that we detect. Further, many lineages may have diversified in response to specific climatic changes, such as Artiodactyla in response to Oligocene cooling events.

Our results are consistent with the tropics, globally, being both a museum and a cradle for mammalian diversity, a pattern that is now being demonstrated across groups [17,57]. For Asia too, our results suggest that the tropics act as both cradles and museums of diversity. This implies that the tropics have been accumulating diversity faster and losing it slower, resulting in spatial gradients in species diversity. Moreover, temporal effects such as the age of the tropical biomes, ages of clades (both time-for-speciation effects) and temporal changes in diversification rates can also modulate the rate of accumulation of species between the tropics and the non-tropics resulting in the observed diversity gradients.

Species diversity patterns in Asia are more complex than immediately apparent. Although the diversity patterns are dominated by the tropical biomes, the other biomes demonstrate noteworthy patterns as well. The process of community assembly (speciation, extinction or colonization) is probably different across biomes and across orders. Of particular interest is the Himalayan range, and the regions surrounding it, and the Tibetan plateau. For many orders, these regions show distinct patterns of PD, thus reflecting distinct patterns through which biodiversity has come to be assembled here [58]. Additionally, many species distributed in the Himalayan ranges and the Tibetan plateau may have undergone range contractions and expansions during the last glacial maxima, which is reflected by PD.

## Conclusions

In conclusion, patterns of species and phylogenetic diversity in Asia vary across the tropical and the non-tropical biomes. These dissimilarities are generated by differences in the evolutionary (diversification) histories of lineages across regions. Variations in evolutionary rates (e.g., speciation, extinction) are important in generating these patterns. We also observe differences in rates between mammalian orders, with some showing higher diversifications rates at lower latitudes, and some others at higher latitudes. While it is imperative to conserve tropical landscapes, which generate a lot of diversity, our results suggest that it is also important to conserve regions of relatively low diversity as they may contain assemblages that have unique evolutionary histories.

## Additional file

Additional file 1: **Supplementary Materials Tamma & Ramakrishnan.** DOC document with embedded figures and tables. Few additional figures supporting and furthering the results obtained, along with tables.

## Abbreviations

SR: Species richness
PD: Phylogenetic diversity
Mya: Million years ago
LTT: Lineage through time
