# Supplementary material for: Higher speciation and lower extinction rates influence mammal diversity gradients in Asia
Source: BMC Evol Biol. 2015 Feb 4;15:11. doi: 10.1186/s12862-015-0289-1 (PMC4333168; doi:10.1186/s12862-015-0289-1)
Supplement: Supplementary file 1 — Supplementary Materials Tamma & Ramakrishnan. DOC document with embedded figures and tables. Few additional figures supporting and furthering the results obtained, along with tables. [file 12862_2015_289_MOESM1_ESM.pdf]

Supplementary Information:

Title: Higher speciation and Lower extinction influence mammal diversity gradients in Asia

Authors: Krishnapriya Tamma and Uma Ramakrishnan

1. How Asian are the Asian species?

There are 457 mammalian genera that have distribution in Asia. There are 1863 species of mammals in Asia [1]. If all species in each of these genera, irrespective of their geographic distribution is considered, there are 2549 species. Thus overall, Asian genera have 25% of their species outside the Asian boundaries as defined by this study.

The figure shows the distribution of different proportions of Asian species across the 457 genera. Most genera have species that are restricted in Asia, while few have more than 50% of their species distributed outside this study's definition of Asia. Thus, we consider this dataset to be reflective of processes occurring in Asia and to be an accurate representation of the same.

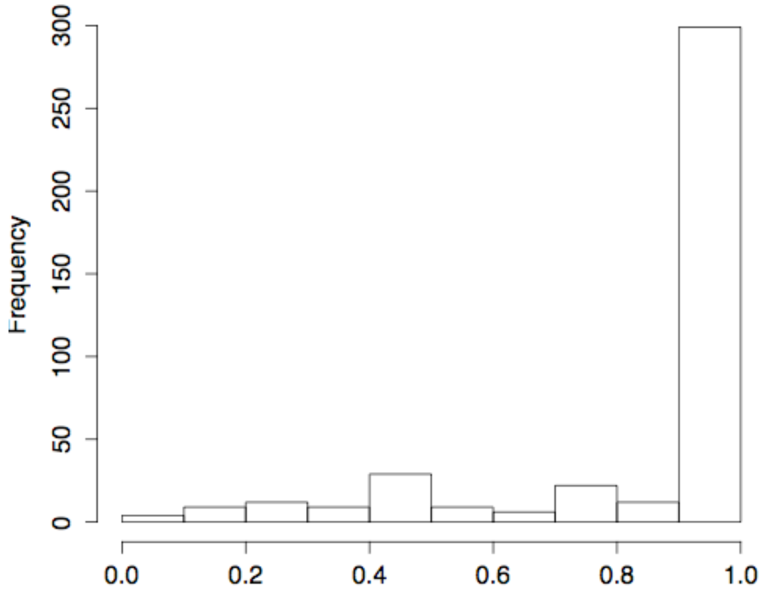

Figure 1: Distribution of the proportion of Asian species across the 457 genera considered in this study that have predominantly Asian presence.

2. How do missing species affect the patterns?

We have three analysis that rely on the phylogeny heavily – Phylogenetic diversity, LTT and Stadler's method for temporal patterns and GeoSSE for spatial patterns. Both Stadler's algorithm and the GeoSSE method can account for missing species while estimating diversification rates.

In total, 405 mammal species are not represented in the supertree. Of these, 275 have geographical ranges described by the IUCN (which is the source of range-data in our study). The remaining 129 have no range data described in the IUCN. Of the 275 species whose ranges are described in the IUCN, 75 species have ranges in Asia.

The 75 species include 1 Monotreme, 6 marsupials, 2 primates, 3 lagomorphs, 2 Erinaceomorphs, 14 soricomorphs, 21 bats, 6 Artiodactyls and 20 rodents. Most of these species are from South and Southeast Asia and Western mountains of the Himalayan system (Supplementary Figure 2). Of the 129 species with no range data in IUCN, 9 are from Asia (whose locations fall within our study area). Most of these species have gone extinct.

Figure 2: South and Southeast Asia (Biomes 1 and 2) along with Biome 4 show the most number of mammal species with missing data. These regions already show the highest phylogenetic diversity in Asia, and adding these missing species to the mammal supertree will only make the patterns of species and phylogenetic diversity recovered by the study only more conspicuous/prominent.

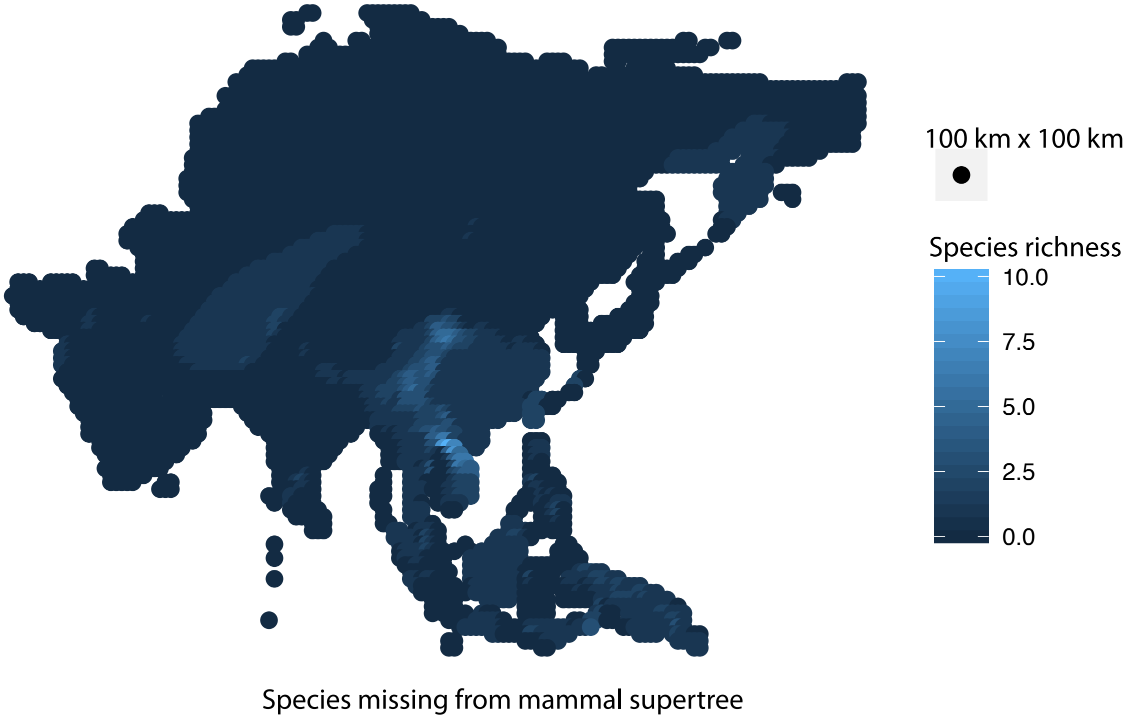

Figure 2: Species richness of missing taxa in Asia

3. How does unrecognized or unknown diversity impact the patterns?

Previous studies have predicted that the highest number of undiscovered mammal species will be from the tropical forests [2]. Given this, and the fact that there have been new species of rodents and shrews described from tropical forests in Asia, it is conceivable that a high proportion of unsampled diversity is hidden tropical regions. Some of the recently discovered mammal species from these regions include - *Leonastus aenigmamus* (striped rabbit) [3], *Saxatilomys pailinae* [4], *Tonkinomys daovantiens* [5], *Hylomys megalotis*, *Chodsigoa caovansunga* [6], *Crocidura kegoensis,* among others [7]. Accounting for this 'hidden' diversity will only accentuate the patterns we observe.

Most this hidden diversity is non-randomly distributed across clades and space. Small mammal orders - such as Chiroptera, Rodentia and Sorcimorpha are witnessing high species discovery. Apart from genetic species, new morpho-species are also being described. Many regions of tropical Asia still remain poorly surveyed and the diversity poorly documented. Species and phylogenetic diversity of this region will increase, and the estimates of diversification rates may subsequently increase for the tropical biome. Given that for Rodentia, Chiroptera and Soricomorpha we recovered higher speciation rates in the tropical biomes, discovery of new species will only increase the estimates of speciation rates.

4. What is the relationship between species richness (SR) and Phylogenetic diversity (PD)?

Figure 3: The relationship between PD and SR for all mammals in Asia. The quadratic spatial regression model best fit the relationship.

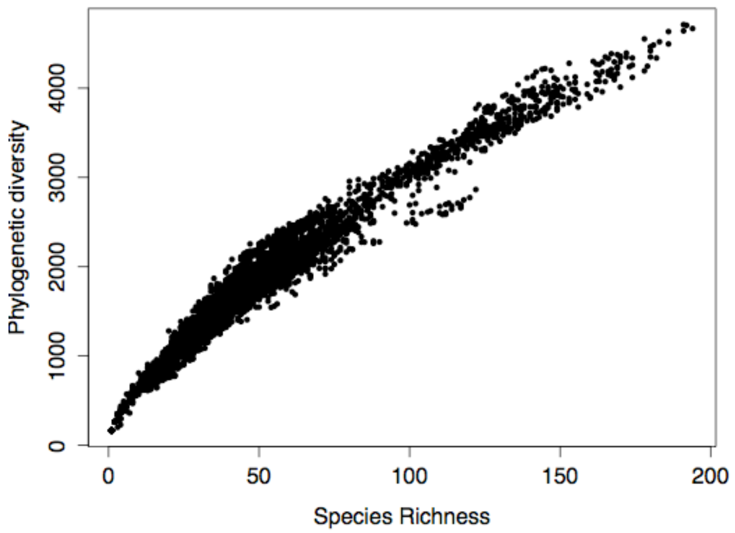

5. What is the relationship between SR and PD for different orders and across biomes?

Figure 4: Panel showing the relationship between PD and SR for different orders across biomes. Each point represents a cell, and the colour denotes the biome it belongs to.

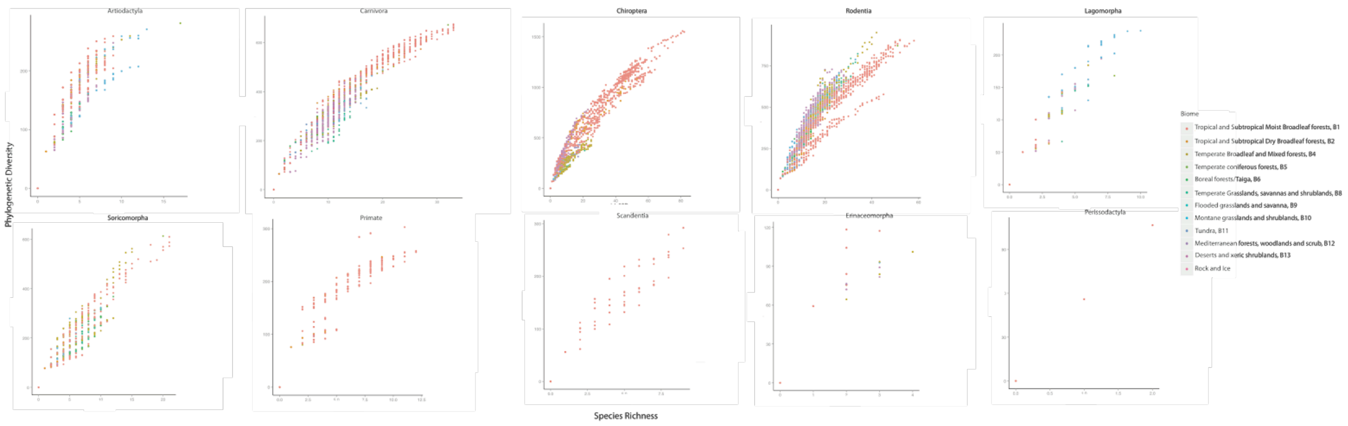

6. Pull of the present and LTT plots:

Inferring speciation and extinction rates solely from LTT plots may be incorrect as they suffer from the 'pull of the present' effects. The pull of the present refers to the apparent increase in diversification rate towards the present, arising from the fact that lineages that have arisen recently are less likely to go extinct and thus are represented in the phylogeny. In our study, we do not estimate diversification rate changes from the LTT, and use the LTT plots as indicators of the overall temporal patterns of lineage diversifications. The diversification shift-times were estimated using the method developed by Stadler et al 2011, which is unaffected by the 'pull of the present'.

7. LTT plots for each of the orders

Figure 5: LTT plots for the individual mammalian orders in Asia.

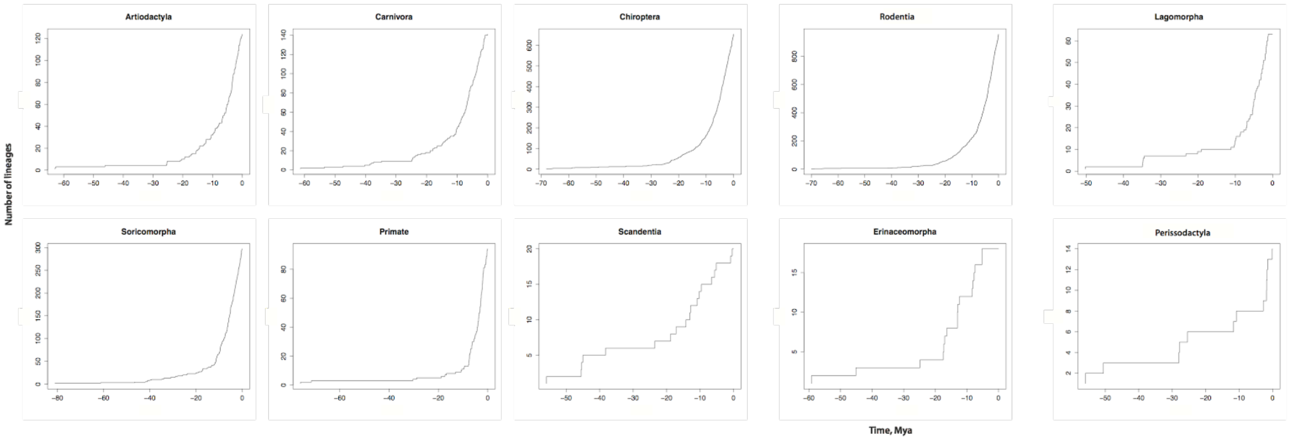

8. What are the patterns of species richness for each of the orders?

Figure 6: Species Richness per order. The lighter shades of blue depict higher species richness and the darker shades depict lower species richness.

a) Artiodactyla, b) Carnivora, c) Chiroptera, d) Lagomorpha, e) Primates, f) Rodentia, and g) Soricomorpha

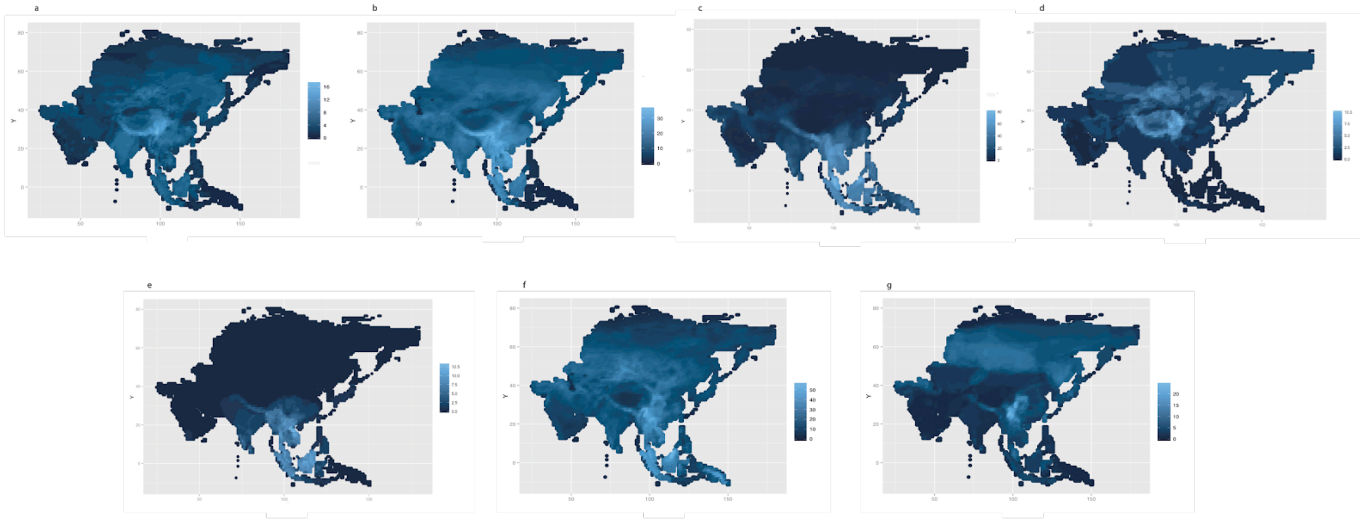

9. Calculation of Sampling frequency:

For all global mammals:

The GeoSSE algorithm can account for missing species by incorporating the sampling frequency of the phylogeny in the likelihood estimations. Sampling frequency refers to the proportion of the species in each geographic region present in our phylogeny. Out of the total of

5416 global mammals, 3959 species were included in our phylogeny. This included 2718 which belonged to both regions, 638 that belonged to tropical biomes and 603 that belonged to non-tropical biomes. Initially, we able to ascribe geographic state for 3038 species belonging to both regions, 804 belonging to tropical regions and 671 belonging to non-tropical regions. We then estimated the sampling frequencies to be as follows: 0.741 for species spanning both biome types, 0.66 for species restricted in the tropical biomes and 0.747for species restricted to the non-tropical biomes.
E.g., Sampling frequency, for species belonging to both biomes = 2718 / (5416 * 3038 / 4505)

For all Asian mammals:
Out of the total of 2549 mammals that had Asian affinities or sisters distributed in Asia (of which 1863 are restricted to Asia), 1670 species were included in our phylogeny. 75 out of 1863 species have no genetic data and hence are excluded from the mammal supertree. The 1670 species included included 782, which belonged to both regions, 488 that belonged to tropical biomes, and 394 that belonged to non-tropical biomes. Initially, we were able to ascribe geographic state for 841 species belonging to both regions, 578 belonging to tropical regions and 432 belonging to non-tropical regions. To calculate the sampling frequency, we did consider the total species to be 2549 in order to incorporate the entire evolutionary history of every genus found in Asia (that is the complete monophyletic lineage). We thus estimated the sampling frequencies to be as follows: 0.67 for species spanning both biome types, 0.613 for species restricted in the tropical biomes and 0.662 for species restricted to the non-tropical biomes.
The inclusion of all species in a genus even if they were not distributed in Asia was inevitable to take into consideration the entire evolutionary history of the genus. If we ignored some species in every genus, it would be an underestimate of the number of evolutionary events that have occurred in the genus, thus resulting in lower speciation estimates. But this results in a decrease in the sampling frequency fed into the algorithm, leading the algorithm to assume that the missing species are also found in the corresponding biomes in Asia. However, given that most sister-species pairs show similar niches, it is conceivable that the species outside of Asia also belong to similar biomes as their sister species in Asia. Thus, the diversifications rates estimated by our method should be an accurate representation of the true trends, while the estimates themselves may have some inaccuracies.

Table 1: Table showing the strength of the relationship between PD and SR regressions for all mammals and across different orders.

Taxa	R2	p value
All mammals	0.985	< 0.05
Rodents	0.963	< 0.05
Carnivora	0.973	< 0.05
Artiodactyla	0.935	< 0.05
Chiroptera	0.985	< 0.05
Soricomorpha	0.947	< 0.05
Lagomorpha	0.949	< 0.05
Primates	0.97	< 0.05

Table 2: The estimates of shift times for the Kuhn-tree

Clade	Number of significant shifts	Shift Time, Mya
Rodentia	1	40
Artiodactyla	1	30
Chiroptera	1	25
Soricomorpha	1	5
Lagomorpha	2	15, 40
Erinaceomorpha	1	5
Scandentia	1	25
Carnivora	0	-
Perrisodactyla	0	-
Pholidota	0	-
Primate	0	-

Table 3: The 16 scenarios considered in this study for the GeoSSE models

**Constraints**	**Interpretation**	**Constraint on speciation, extinction and dispersal rates**	**Speciation by biome divergence**
Full model	No constraints, all parameters can vary	No constraints	No constraints
sAB =! 0	All vary, except speciation by biome divergence is constrained to be not zero	Speciation, extinction and dispersal can vary	Not zero, ranges can split
sA = sB, sAB =! 0	All vary, except spectaion rates in the two biomes are constrained to be equal, and speciation by biome divergence is constrained to be not zero	Extinction and dispersal rates can vary	Not zero, ranges can split
sAB = 0	All vary, except speciation by biome divergence is constrained to be zero	Speciation, extinction and dispersal can vary	Zero
sA = sB, sAB = 0	All vary, except speciation in the biomes is equal and speciation by biome divergence is zero	Extinction and dispersal rates can vary	Zero
sAB =! 0, xA = xB	All vary, except speciation by biome divergence is not zero and extinction in both biomes is equal	Speciation and dispersal rates can vary	Not zero, ranges can split
sAB =! 0, dA = dB	All vary, speciation by biome divergence is not zero and dispersal across biomes is equal	Speciation and extinction rates can vary	Not zero, ranges can split
sAB =! 0, xA = xB, dA = dB	All vary, speciation by biome divergence is not zero, extinction and dispersal across biomes in equal	Speciation rates can vary	Not zero, ranges can split
sAB = 0, xA = xB	All vary, speciation by biome divergence is zero and extinction across the regions is equal	Speciation and dispersal rates can vary	Zero
sA = sB, sAB =! 0, dA = dB	All vary, speciation and dispersal across the both regions is equal and speciation by biome divergence is not zero	Extinction rates can vary	Not zero, ranges can split
sAB = 0, dA = dB	All vary, speciation by biome divergence is zero and dispersal across the biomes is equal	Speciation and extinction rates can vary	Zero
sA = 0, xA = xB, dA = dB	All vary, speciation in region A is zero, extinction and dispersal are equal across the regions	Specation rates can vary	Zero
sA = sB, sAB = 0, dA = dB	All vary, speciation and dispersal across regions is equal, speciation by biome divergence is zero	Extinction rates can vary	Zero
sA = sB, sAB = 0, xA = xB	All vary, speciation and extinction across the regions is equal, speciation by biome divergence is zero	Dispersal rates can vary	Zero
sA = sB, sAB =! 0, xA = xB, dA = dB	All vary, speciation, extinction and dispersal across both regions is equal, speciation by biome divergence is not zero	All constrained	Not zero, ranges can split
sA = sB, sAB = 0, xA = xB, dA = dB	Speciation, extinction and dispersal across the regions is equal and the speciation by	All constrained	Zero

	biome divergence is zero		

Table 4: Estimates of speciation, extinction and dispersal based on GeoSSE for all global mammals (global-tree). The likelihood estimates are presented here.

**Model**	**Constraints**	**sA**	**sB**	**sAB**	**xA**	**xB**	**dA**	**dB**	**AIC**
fitp	Full model	1.58E-01	4.46E-02	1.69E-07	7.72E-02	5.27E-01	2.61E+00	4.47E-03	33103
fit1c	sAB =! 0	1.59E-01	1.45E-05	1.00E+00	1.08E-01	4.46E+00	1.76E+01	1.34E-01	34187
fit2c	sA = sB, sAB =! 0	1.23E-01	1.23E-01	1.00E+00	1.23E-01	5.07E+00	1.94E+01	4.17E-01	34252
**fit3c**	**sAB = 0**	1.58E-01	4.13E-02	0.00E+00	7.64E-02	5.10E-01	2.37E+00	1.52E-03	33101
fit4c	sA = sB, sAB = 0	1.34E-01	1.34E-01	0.00E+00	5.04E-02	6.71E-01	2.75E+00	3.51E-04	33336
fit5c	sAB =! 0, xA = xB	9.76E-09	9.76E-03	1.00E+00	1.27E+00	1.27E+00	4.14E+00	5.79E+00	35273
fit6c	sAB =! 0, dA = dB	1.84E-07	1.06E-02	1.00E+00	8.91E-01	2.05E+00	5.48E+00	5.48E+00	35229
fit7c	sAB =! 0, xA = xB, dA = dB	6.43E-03	3.34E-06	1.00E+00	1.36E+00	1.36E+00	5.44E+00	5.44E+00	35296
fit8c	sAB = 0, xA = xB	0.20972783	0.01719648	0	0.35619925	0.35619925	1.87088505	1.35296867	33160
fit9c	sA = sB, sAB =! 0, dA = dB	0.002962184	0.002962184	1	0.942030942	1.993341146	5.616598083	5.616598083	35228
fit10c	sAB = 0, dA = dB	0.01484798	0.21709661	0	0.22109782	0.38870073	1.28828496	1.28828496	33183
fit11c	sA = 0, xA = xB, dA = dB	0	0.207046308	0.009459933	0.347930179	0.347930179	1.702022631	1.702022631	35271

fit13c	sA = sB, sAB = 0, dA = dB	0.1218341	0.1218341	0	0.3578156	0.4393606	1.8499627	1.8499627	33352
fit14c	sA = sB, sAB = 0, xA = xB	0.1213707	0.1213707	0	0.4128649	0.4128649	1.8093814	2.2319052	33363
fit15c	sA = sB, sAB =! 0, xA = xB, dA = dB	0.003060672	0.003060672	1	1.361310715	1.361310715	5.462827093	5.462827093	33598
fit16c	sA = sB, sAB = 0, xA = xB, dA = dB	0.1205721	0.1205721	0	0.3903344	0.3903344	1.8188365	1.8188365	33351

Table 5: Estimates of speciation, extinction and dispersal based on GeoSSE for all mammals in Asia (Asia-tree). The likelihood estimates are presented here.

**Likelihood models**	**Constrains**	**sA**	**sB**	**sAB**	**xA**	**xB**	**dA**	**dB**		**AIC**
**likp, fitp**		1.30E-01	6.22E-02	2.87E-06	1.16E-05	3.90E-02	2.10E-01	1.27E-02		14341
lik2, fitp2		1.78E-01	1.78E-01	1.00E+00	1.25E-01	2.18E+00	3.77E+00	1.01E-02		15182
**lik3, fitp3**		**1.30E-01**	**6.22E-02**	**0.00E+00**	**1.16E-05**	**3.90E-02**	**2.10E-01**	**1.27E-02**		**14339**
lik4, fitp4		1.13E-01	1.13E-01	0.00E+00	5.14E-05	1.14E-01	2.67E-01	6.85E-03		14415
lik5, fitp5		1.81E-03	1.16E-01	1.00E+00	6.15E-01	6.15E-01	9.39E-01	1.74E+00		15262
lik6, fitp6		2.77E-06	1.53E-01	1.00E+00	4.38E-01	1.01E+00	1.44E+00	1.44E+00		15246
lik7, fitp7		0.11723122	0.01332264	1	0.63676074	0.63676074	1.29685155	1.29685155		15284
lik8, fitp8		1.34E-01	4.75E-02	0.00E+00	3.25E-08	3.25E-08	1.64E-01	1.93E-02		14390
lik9, fitp9		5.51E-02	5.51E-02	1.00E+00	4.98E-01	8.76E-01	1.44E+00	1.44E+00		15261
lik10, fitp10		0.15994	0.06240	0	0.13923	0.09077	0.21011	0.21011		15261

		94	336		937	896	332	332		
lik11, fitp11		0	0.2424	0.1412596	0.4229049	0.4229049	1.0011564	1.0011564		14654
lik12, fitp12		0.05544669	0.05544669	1	0.6437525	0.6437525	1.12562296	1.70175537		14990
lik13, fitp13		0.1006649	0.1006649	0	0.06397932	0.07322679	0.15071591	0.15071591		14720
lik14, fitp14		0.1021089	0.1021089	0	0.07148	0.07148	0.142857	0.1635954		14723
lik15, fitp15		0.0562133	0.0562133	1	0.6462286	0.6462286	1.3775217	1.3775217		15294
lik16, fitp16		0.09889181	0.09889181	0	0.05294321	0.05294321	0.13056265	0.13056265		14731

Table 6: Global and Asia: Summary of best models for each order based on analyses from trees from two trees - Rolland et al and Kuhn et al trees. Additionally also for Nyakatura tree for Carnivores.

**Order**	**Model**	**Phylogenetic tree**	**sA**	**sB**	**sAB**	**xA**	**xB**	**dA**	**dB**
**All Global Mammals**		Rolland	1.58E-01	4.13E-02	0.00E+00	7.64E-02	5.10E-01	2.37E+00	1.52E-03
		Kuhn	1.44E-01	3.44E-02	0.00E+00	6.74E-02	4.27E-01	2.08E+00	1.96E-06
**All Asian Mammals**		Rolland	1.30E-01	6.22E-02	0	1.16E-05	3.90E-02	2.10E-01	1.20E-02
		Kuhn	1.20E-01	6.19E-02	0.00E+00	1.36E-09	4.58E-02	2.11E-01	1.04E-02
**Rodents**		Rolland	1.53E-01	9.19E-02	0.00E+00	4.72E-09	4.01E-02	1.72E-01	9.14E-03
		Kuhn	1.29E-01	8.73E-02	0.00E+00	7.81E-09	4.86E-02	1.58E-01	6.96E-03
**Soricomorpha**		Rolland	1.39E-01	6.26E-02	4.12E-03	7.43E-09	5.23E-09	1.22E-01	1.89E-02
		Kuhn	1.35E-01	6.55E-02	9.52E-04	1.95E-07	1.30E-02	1.40E-01	1.64E-02
**Chiroptera**		Rolland	1.64E-01	4.61E-02	0.00E+00	7.62E-02	4.49E-01	9.80E-01	2.23E-07
		Kuhn	1.64E-01	3.73E-02	0.00E+00	8.28E-02	4.45E-01	9.96E-01	4.49E-07

**Carnivora**	4	Rolland	0.07998084	0.07998084	0	0.52548065	0.21761019	4.13032025	2.25580311
		Kuhn	0.07301572	0.07301572	0	0.23903662	0.2125223	3.91219395	1.00811563
		Nyakatura	0.1115864	0.1115864	0	0.2753405	0.6017665	8.6906934	0.8564661
	13	Rolland	0.08068592	0.08068592	0	0.68228871	0.16660667	2.95147745	2.95147745
		Kuhn	0.07599355	0.07599355	0	0.61291447	0.14958541	2.64040214	2.64040214
		Nyakatura	0.1145309	0.1145309	0	1.3038101	0.3820099	5.2220127	5.2220127
	14	Rolland	0.07854467	0.07854467	0	0.25458782	0.25458782	4.74608909	1.06121952
		Kuhn	0.07377427	0.07377427	0	0.22821546	0.22821546	4.25907653	0.96021213
		Nyakatura	1.15E-01	1.15E-01	0.00E+00	1.30E+00	3.82E-01	5.22E+00	5.22E+00
**Artiodactyla**	3	Rolland	8.80E-02	1.30E-01	0.00E+00	6.74E-02	2.13E-08	1.71E-02	2.61E-01
		Kuhn	2.43E-01	7.33E-07	0.00E+00	2.06E-01	4.45E-01	1.09E+00	2.31E-06
	4	Rolland	1.17E-01	1.17E-01	0.00E+00	1.03E-01	9.14E-08	1.54E-02	2.92E-01
		Kuhn	1.48E-01	1.48E-01	0.00E+00	8.68E-02	4.01E-01	8.28E-01	7.69E-07
	8	Rolland	6.49E-02	1.27E-01	0.00E+00	2.25E-09	2.25E-09	2.92E-02	1.45E-01
		Kuhn	2.86E-01	1.32E-08	0.00E+00	3.29E-01	3.29E-01	8.48E-01	3.00E-01

Table 7: List of Asian genera

Sl No	Genus
1	Ursus
2	Rangifer
3	Dicrostonyx
4	Canis
5	Mustela

6	Lepus
7	Lemmus
8	Gulo
9	Microtus
10	Ochotona
11	Sorex
12	Vulpes
13	Rattus
14	Sciurus
15	Alces
16	Myodes
17	Mus
18	Muscardinus
19	Musonycteris
20	Alticola
21	Pteromyscus
22	Pteromys
23	Lynx
24	Myopus
25	Marmota
26	Ovis
27	Arvicola
28	Martes
29	Ovibos
30	Talpa
31	Tamias
32	Tamiasciurus
33	Spermophilus
34	Moschus
35	Apodemus
36	Sicista
37	Meles
38	Micromys

39	Eptesicus
40	Capreolus
41	Myotis
42	Lutra
43	Plecotus
44	Neomys
45	Erinaceus
46	Vespertilio
47	Castor
48	Cricetus
49	Crocidura
50	Cervus
51	Murina
52	Desmana
53	Lagurus
54	Sus
55	Myospalax
56	Meriones
57	Allactaga
58	Ellobius
59	Phodopus
60	Cricetulus
61	Mesechinus
62	Cuon
63	Eliomys
64	Allocricetulus
65	Hemiechinus
66	Nyctalus
67	Enhydra
68	Panthera
69	Nyctereutes
70	Vormela
71	Capra

72	Pipistrellus
73	Stylodipus
74	Dipus
75	Tscherskia
76	Pygeretmus
77	Saiga
78	Cardiocranius
79	Lasiopodomys
80	Diplomesodon
81	Spalax
82	Dryomys
83	Eolagurus
84	Gazella
85	Procapra
86	Prionailurus
87	Rhombomys
88	Salpingotus
89	Mogera
90	Allactodipus
91	Selevinia
92	Felis
93	Equus
94	Naemorhedus
95	Eremodipus
96	Spermophilopsis
97	Arctonyx
98	Scaptochirus
99	Caracal
100	Euchoreutes
101	Camelus
102	Niviventer
103	Miniopterus
104	Nesokia

105	Barbastella
106	Rhinolophus
107	Prometheomys
108	Chionomys
109	Glis
110	Glischropus
111	Rupicapra
112	Otonycteris
113	Hystrix
114	Paradipus
115	Tadarida
116	Brachiones
117	Eospalax
118	Mesocricetus
119	Jaculus
120	Suncus
121	Hyaena
122	Paraechinus
123	Mellivora
124	Sciurotamias
125	Blanfordimys
126	Neodon
127	Pseudois
128	Paguma
129	Trogopterus
130	Capricornis
131	Dymecodon
132	Macaca
133	Petaurista
134	Euroscaptor
135	Chimarrogale
136	Glirulus
137	Urotrichus

138	Herpestes
139	Myomimus
140	Calomyscus
141	Phaiomys
142	Przewalskium
143	Taphozous
144	Tamiops
145	Chodsigoa
146	Aeretes
147	Hydropotes
148	Bos
149	Boselaphus
150	Eozapus
151	Caryomys
152	Elaphurus
153	Damaliscus
154	Dama
155	Rousettus
156	Gerbillus
157	Tatera
158	Asellia
159	Acinonyx
160	Eupetaurus
161	Pantholops
162	Cansumys
163	Scapanulus
164	Acomys
165	Psammomys
166	Rhinopoma
167	Semnopithecus
168	Eoglaucomys
169	Hyperacrius
170	Pteropus

171	Blarinella
172	Eothenomys
173	Rhizomys
174	Myocastor
175	Procavia
176	Megaderma
177	Scotophilus
178	Kerivoula
179	Bandicota
180	Pardofelis
181	Muntiacus
182	Rhinopithecus
183	Proedromys
184	Vernaya
185	Ia
186	Nectogale
187	Callosciurus
188	Belomys
189	Nycteris
190	Hipposideros
191	Funambulus
192	Golunda
193	Manis
194	Hemitragus
195	Rusa
196	Viverra
197	Anourosorex
198	Leopoldamys
199	Scaptonyx
200	Ailurus
201	Budorcas
202	Elaphodus
203	Viverricula

204	Melogale
205	Uropsilus
206	Dremomys
207	Ailuropoda
208	Typhlomys
209	Cynopterus
210	Scotoecus
211	Scotozous
212	Millardia
213	Paradoxurus
214	Neofelis
215	Volemys
216	Chaetocauda
217	Oryx
218	Lutrogale
219	Axis
220	Vandeleuria
221	Prionodon
222	Neotetracus
223	Hylopetes
224	Scotomanes
225	Sekeetamys
226	Melursus
227	Aonyx
228	Sphaerias
229	Falsistrellus
230	Soriculus
231	Episoriculus
232	Parascaptor
233	Tylonycteris
234	Atherurus
235	Coelops
236	Berylmys

237	Triaenops
238	Salpingotulus
239	Tetracerus
240	Rucervus
241	Elephas
242	Eonycteris
243	Tupaia
244	Trachypithecus
245	Cremnomys
246	Antilope
247	Moschiola
248	Caprolagus
249	Rhinoceros
250	Megaerops
251	Arctictis
252	Ratufa
253	Helarctos
254	Aselliscus
255	Hesperoptenus
256	Philetor
257	Dacnomys
258	Arielulus
259	Cannomys
260	Macroglossus
261	Harpiocephalus
262	Nycticebus
263	Arctogalidia
264	Bubalus
265	Biswamoyopterus
266	Chiropodomys
267	Tokudaia
268	Pentalagus
269	Diplothrix

270	Diomys
271	Petinomys
272	Ichneumia
273	Saccolaimus
274	Otomops
275	Hadromys
276	Hylomyscus
277	Hylomys
278	Nomascus
279	Hapalomys
280	Anathana
281	Madromys
282	Menetes
283	Genetta
284	Hylobates
285	Chiromyscus
286	Chrotogale
287	Maxomys
288	Tragulus
289	Eudiscopus
290	Phoniscus
291	Dendrogale
292	Galeopterus
293	Eidolon
294	Myomyscus
295	Pygathrix
296	Nesolagus
297	Paracoelops
298	Neohylomys
299	Acerodon
300	Emballonura
301	Otopteropus
302	Ptenochirus

303	Haplonycteris
304	Cheiromeles
305	Dyacopterus
306	Phloeomys
307	Apomys
308	Bullimus
309	Rhynchomys
310	Batomys
311	Archboldomys
312	Carpomys
313	Chrotomys
314	Crunomys
315	Papio
316	Craseonycteris
317	Tryphomys
318	Abditomys
319	Crateromys
320	Tapirus
321	Loris
322	Arvicanthis
323	Platacanthomys
324	Hemigalus
325	Harpyionycteris
326	Cynogale
327	Presbytis
328	Styloctenium
329	Anonymomys
330	Exilisciurus
331	Sundasciurus
332	Cynocephalus
333	Tarsius
334	Feroculus
335	Sundamys

336	Echinosorex
337	Mydaus
338	Haeromys
339	Nyctimene
340	Dobsonia
341	Latidens
342	Podogymnura
343	Urogale
344	Rhinosciurus
345	Lariscus
346	Palawanomys
347	Alionycteris
348	Tarsomys
349	Limnomys
350	Srilankamys
351	Balionycteris
352	Aeromys
353	Chironax
354	Solisorex
355	Penthetor
356	Ptilocercus
357	Petaurillus
358	Rheithrosciurus
359	Trichys
360	Aethalops
361	Symphalangus
362	Nannosciurus
363	Iomys
364	Lenothrix
365	Pithecheir
366	Dicerorhinus
367	Pongo
368	Diplogale

369	Glyphotes
370	Nasalis
371	Mormopterus
372	Melomys
373	Ailurops
374	Thoopterus
375	Prosciurillus
376	Strigocuscus
377	Phalanger
378	Mosia
379	Macrogalidia
380	Taeromys
381	Lenomys
382	Paruromys
383	Bunomys
384	Rubrisciurus
385	Echiothrix
386	Margaretamys
387	Petaurus
388	Syconycteris
389	Babyrousa
390	Hyosciurus
391	Neopteryx
392	Dendrolagus
393	Dactylopsila
394	Echymipera
395	Spilocuscus
396	Uromys
397	Myoictis
398	Paranyctimene
399	Paramelomys
400	Dasyurus
401	Hydromys

402	Pseudochirulus
403	Cercartetus
404	Xenuromys
405	Peroryctes
406	Pseudochirops
407	Zaglossus
408	Simias
409	Melasmothrix
410	Tateomys
411	Eropeplus
412	Dorcopsis
413	Nyctophilus
414	Pogonomelomys
415	Phascolosorex
416	Distoechurus
417	Pogonomys
418	Hyomys
419	Murexia
420	Microperoryctes
421	Parahydromys
422	Thylogale
423	Mammelomys
424	Paraleptomys
425	Nesoromys
426	Rhynchomeles
427	Neophascogale
428	Anisomys
429	Mallomys
430	Dorcopsulus
431	Microhydromys
432	Macruromys
433	Lorentzimys
434	Pseudohydromys

435	Coccymys
436	Crossomys
437	Sminthopsis
438	Aproteles
439	Abeomelomys
440	Protochromys
441	Tachyglossus
442	Leptomys
443	Melonycteris
444	Chiruromys
445	Scotorepens
446	Kadarsanomys
447	Macropus
448	Chalinolobus
449	Komodomys
450	Papagomys
451	Planigale
452	Isoodon
453	Pseudomys
454	Lagorchestes
455	Conilurus
456	Xeromys
457	Pharotis

References

1. Wilson DE, Reeder DM: *Mammal Species of the World*. 3rd edition. Baltimore: Johns Hopkins University Press; 2005:2142.

2. Giam X, Scheffers BR, Sodhi NS, Wilcove DS, Ceballos G, Ehrlich PR: **Reservoirs of richness: least disturbed tropical forests are centres of undescribed species diversity.** *Proc Biol Sci* 2012, **279**:67–76.

3. Surridge AK, Timmins RJ, Hewitt GM, Bell DJ: **Striped rabbits in Southeast Asia**. *Nature* 1999, **400**(August):1999.

4. Musser GG, Smith AL, Robinson MF, Lunde DP: **Description of a New Genus and Species of Rodent (Murinae , Muridae , Rodentia) from the Khammouan Limestone National Biodiversity Conservation Area in Lao PDR**. *Am museum Novit* 2005, **29412**:1–32.

5. Musser GUYG, Lunde DP, Son NT: **Description of a New Genus and Species of Rodent (Murinae , Muridae , Rodentia) from the Tower Karst Region of Northeastern Vietnam**. *Am Museum Novit* 2006.

6. Lunde DP, Musser GG, Son NT: **A survey of small mammals from Mt . Tay Con Linh II , Vietnam , with the description of a new species of Chodsigoa (Insectivora : Soricidae)**. *Mammal Study* 2003, **46**:31–46.

7. Jenkins PD, Abramov A V, Bannikova A a, Rozhnov V V: **Bones and genes: resolution problems in three Vietnamese species of Crocidura (Mammalia, Soricomorpha, Soricidae) and the description of an additional new species.** *Zookeys* 2013, **79**:61–79.
